# Endoscopic drainage for a thoracic abscess caused by delayed perforation after endoscopic submucosal dissection for gastric tube cancer: a case report

**DOI:** 10.1055/a-2568-7596

**Published:** 2025-04-29

**Authors:** Aiji Hattori, Yuji Owaki, Atsushi Inaba, Daisuke Kajiyama, Kazuma Sato, Takeo Fujita, Tomonori Yano

**Affiliations:** 1 Department of Gastroenterology and Endoscopy, National Cancer Center Hospital East, Chiba, Japan; 2 Department of Esophageal Surgery, National Cancer Center Hospital East, Chiba, Japan

A 75-year-old man with a history of esophageal cancer treated with subtotal esophagectomy developed a 10-mm early gastric tube cancer (GTC) ( Fig. 1 ). Endoscopic submucosal dissection (ESD) was performed without immediate complications, and he was discharged six days later. Four days post-discharge, the patient presented with fever and lower back pain. Computed tomography (CT) revealed gastric wall thinning and a thoracic abscess in the right lower lung field ( Fig. 2 ). We diagnosed the patient with delayed perforation after ESD with a thoracic abscess. We initiated thoracic drainage, nasogastric tube insertion, and antibiotics.

**Fig. 1:**
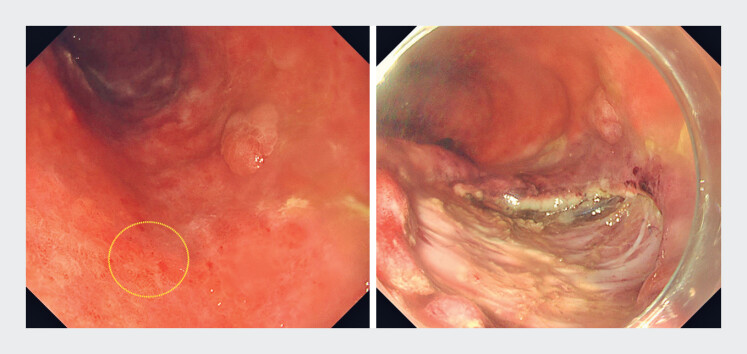
A 10-mm early gastric tube cancer was identified on the posterior wall of the middle gastric tube (illustrated by the circle). Endoscopic submucosal dissection was performed on the lesion.

**Fig. 2:**
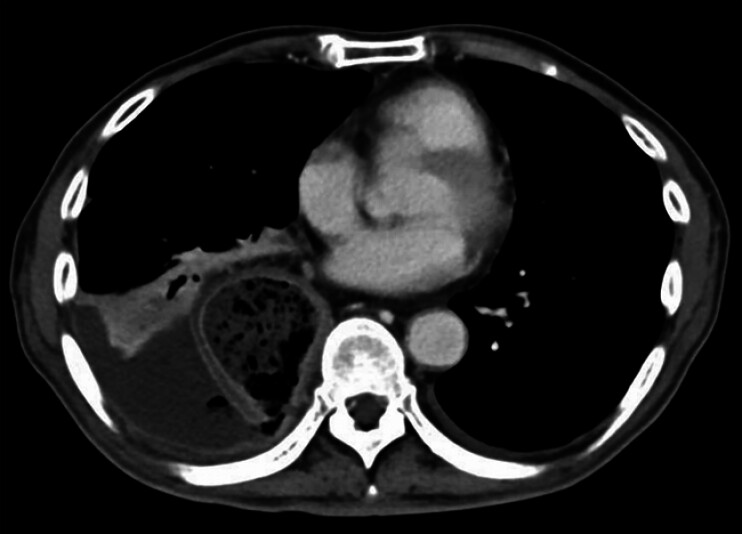
Computed tomography (CT) revealed the presence of gastric tube contents, a reduction in the thickness of the gastric tube wall as well as the presence of pleural effusion in the right posterior lower lung field.

Despite the initial interventions, his symptoms did not improve. An endoscopic examination was performed to assess the perforation site and revealed food residue in the gastric tube, which was removed. We attempted fistula closure with clips but failed due to mucosal edema and fibrosis ( Fig. 3 ). With persistent symptoms and inadequate drainage on follow-up CT, we attempted endoscopic drainage collaborating with thoracic surgeons.

**Fig. 3:**
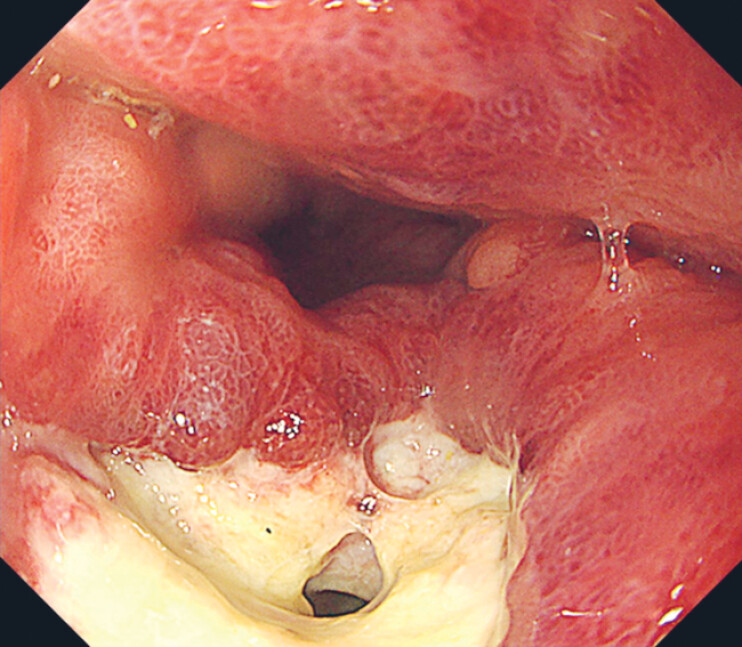
The locus of perforation was duly recognized. The attempted closure of the fistula utilizing clips was deemed unsuccessful owing to considerable edema and fibrotic tissue surrounding the ulcer.

An ultrathin endoscope was inserted directly through the fistula into the abscess cavity for washing, removing septa within the abscess, and placing the guidewire, followed by nasogastric tube placement near the thoracic drain tube to make the communication ( Video 1 , Fig. 4 ).

Direct endoscopic drainage for a thoracic abscess caused by delayed perforation after endoscopic submucosal dissection for gastric tube cancer.Video 1

**Fig. 4:**
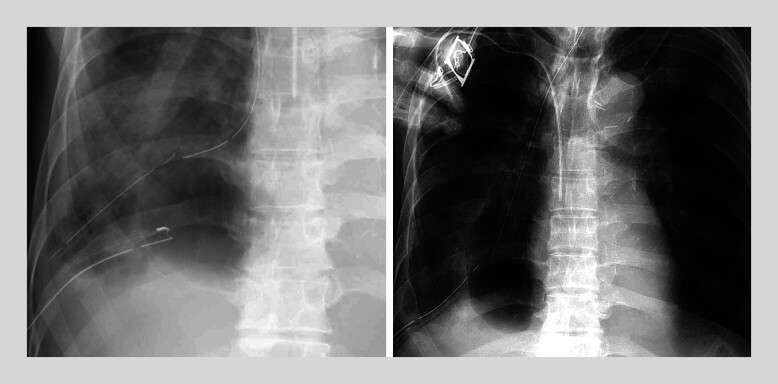
A nasogastric tube was placed near the thoracic drain tube to make the communication.

Continuous saline irrigation led to abscess shrinkage ( Fig. 5 ), symptom improvement, and discharge at 35 days post-admission.

**Fig. 5:**
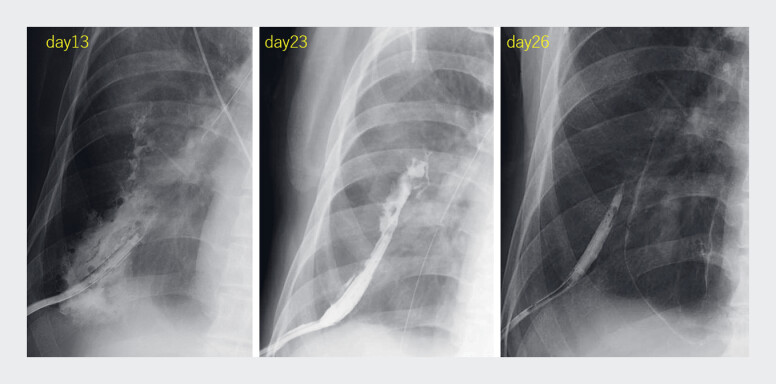
Following the procedures, systematic saline irrigation was conducted on a daily basis via the nasogastric tube, resulting in a progressive reduction in the size of the abscess.

Only four cases of delayed perforation after ESD for GTC have been reported, all diagnosed early (1–3 days) post-ESD and treated conservatively 1 2 3 4 . In this case, the perforation was diagnosed 10 days after ESD. As time passed after the perforation, endoscopic closure of the fistula became challenging because of edema and fibrosis of the mucosa. Surgery for delayed perforation of GTC is highly invasive and difficult owing to its adhesion to vital organs. We performed direct endoscopic abscess drainage as an effective conservative treatment to avoid surgery.

Endoscopy_UCTN_Code_CPL_1AH_2AZ_3AD

## Notes

Endoscopy E-Videos https://eref.thieme.de/e-videos
*E-Videos* is an open access online section of the journal *Endoscopy* , reporting on interesting cases and new techniques in gastroenterological endoscopy. All papers include a high-quality video and are published with a Creative Commons CC-BY license. Endoscopy E-Videos qualify for HINARI discounts and waivers and eligibility is automatically checked during the submission process. We grant 100% waivers to articles whose corresponding authors are based in Group A countries and 50% waivers to those who are based in Group B countries as classified by Research4Life (see: https://www.research4life.org/access/eligibility/ ). This section has its own submission website at https://mc.manuscriptcentral.com/e-videos .
